# Factors influencing the development of otitis media among Sicilian children affected by upper respiratory tract infections^☆^

**DOI:** 10.1016/j.bjorl.2015.04.002

**Published:** 2015-07-21

**Authors:** Francesco Martines, Pietro Salvago, Sergio Ferrara, Giuseppe Messina, Marianna Mucia, Fulvio Plescia, Federico Sireci

**Affiliations:** a Section of Otolaryngology, Department of Experimental Biomedicine and Clinical Neurosciences, Università degli Studi di Palermo, Palermo, Italy; b Section of Audiology, Department of Biotechnology and Medical and Forensic Biopathology, Università degli Studi di Palermo, Palermo, Italy; c Department of Science for the Promotion of Health, Università degli Studi di Palermo, Palermo, Italy; d Sport and Exercise Sciences Research Unit, Università degli Studi di Palermo, Palermo, Italy

**Keywords:** Otitis media, URTI, Risk factors, Otite média, IVAS, Fatores de risco

## Abstract

**Introduction:**

Upper respiratory tract infection is a nonspecific term used to describe an acute infection involving the nose, paranasal sinuses, pharynx and larynx. Upper respiratory tract infections in children are often associated with Eustachian tube dysfunction and complicated by otitis media, an inflammatory process within the middle ear. Environmental, epidemiologic and familial risk factors for otitis media (such as sex, socioeconomic and educational factors, smoke exposure, allergy or duration of breastfeeding) have been previously reported, but actually no data about their diffusion among Sicilian children with upper respiratory tract infections are available.

**Objective:**

To investigate the main risk factors for otitis media and their prevalence in Sicilian children with and without upper respiratory tract infections.

**Methods:**

A case–control study of 204 children with upper respiratory tract infections who developed otitis media during a 3 weeks monitoring period and 204 age and sex-matched healthy controls. Seventeen epidemiologically relevant features were inventoried by means of standardized questionnaires and skin tests were performed. Univariate analysis and multivariate logistic regression analysis were used to examine the association between risk factors and occurrence of otitis media.

**Results:**

Otitis media resulted strongly associated to large families, low parental educational attainment, schooling within the third years of life (*p* < 0.05); children were more susceptible to develop otitis media in the presence of asthma, cough, laryngopharyngeal reflux disease, snoring and apnea (*p* < 0.05). Allergy and urban localization increased the risk of otitis media in children exposed to smoke respectively of 166% and 277% (*p* < 0.05); the joint effect of asthma and presence of pets in allergic population increased the risk of recurrence of 11%, while allergy, cough and runny nose together increased this risk of 74%.

**Conclusions:**

Upper respiratory tract infections and otitis media are common childhood diseases strongly associated with low parental educational attainment (*p* = 0.0001), exposure to smoke (*p* = 0.0001), indoor exposure to mold (*p* = 0.0001), laryngopharyngeal reflux disease (*p* = 0.0002) and the lack of breast-feeding (*p* = 0.0014); an increased risk of otitis media recurrences was observed in the presence of allergy, persistent cough and runny nose (*p* = 0.0001). The modification of the identified risk factors for otitis media should be recommended to realize a correct primary care intervention.

## Resumo

**Introdução:**

A infecção de vias aéreas superiores é uma denominação inespecífica, empregada na descrição de uma infecção aguda envolvendo o nariz, os seios paranasais, a faringe e a laringe. As infecções de vias aéreas superiores em crianças estão frequentemente associadas à disfunção da trompa de Eustáquio, complicando-se com otite média, um processo inflamatório da orelha média. Já foram relatados vários fatores de risco relacionados à otite média, incluindo os ambientais, epidemiológicos e familiares (p. ex., gênero, fatores socioeconômicos e educacionais, exposição ao tabaco, alergia ou duração do aleitamento materno), entretanto, não dispomos de dados sobre sua ocorrência entre crianças sicilianas com infecções de vias aéreas superiores.

**Objetivo:**

Investigar os principais fatores de risco para otite média e sua prevalência em crianças sicilianas com e sem infecções de vias aéreas superiores.

**Método:**

Um estudo de caso-controle de 204 crianças com infecções de vias aéreas superiores apresentando otite média durante um período de monitoração de três semanas e 204 controles saudáveis compatíveis em idade e gênero. Foram relacionadas 17 características com relevância epidemiológica por meio da aplicação de questionários padronizados; também foram realizados testes cutâneos. Foram utilizadas análises univariada e de regressão logística multivariada no exame da associação entre fatores de risco e ocorrência de otite média.

**Resultados:**

A otite média revelou forte associação com famílias numerosas, baixo nível educacional dos pais e escolaridade no terceiro ano de vida (p< 0,05); as crianças demonstraram maior suscetibilidade para ocorrência de otite média em presença de asma, tosse, doença do refluxo laringofaríngeo, ronco e apneia (p < 0,05). Alergia e localização urbana aumentaram o risco de otite média em crianças expostas ao fumo em 166% e 277% (p < 0,05), respectivamente; o efeito conjunto de asma e presença de animais de estimação na população alérgica aumentou o risco de recidiva em 11%, enquanto que, em conjunto, alergia, tosse e coriza aumentaram esse risco em 74%.

**Conclusões:**

As infecções de vias aéreas superiores e otite média são doenças pediátricas comuns, fortemente associadas a baixo nível educacional dos pais (p = 0,0001), exposição ao fumo (p = 0,0001), exposição domiciliar ao mofo (p = 0,0001), refluxo laringofaríngeo (p = 0,0002) e ausência de aleitamento materno (p = 0,0014). Também foi observado aumento do risco de recidivas de otite média em presença de alergia, tosse persistente e coriza (p = 0,0001). Deve-se recomendar a modificação dos fatores de risco identificados para otite média, para uma correta intervenção terapêutica primária.

## Introduction

Upper respiratory tract infection (URTI) is a non-specific term used to describe an acute infection involving nose, paranasal sinuses, pharynx and larynx.^1^, ^2^ According to World Health Organization (WHO), URTIs are responsible for 20% of annual deaths among children under 5 years of age, and for 13,000 hospitalizations.^3^ URTIs in children are often complicated by otitis media (OM), an inflammatory process within the middle ear, with an incidence ranging from 6% to 64%.^2^, ^4^ OM is classified in two forms: (1) acute OM (AOM), an acute symptomatic disease characterized by earache, fever, hearing impairment and a purulent discharge (otorrhea) through a perforation of the tympanic membrane. (2) OM with effusion (OME), an asymptomatic disease involving fluid collection in the middle ear, associated with either a mild or moderate conductive hearing impairment. Many OM episodes resolve spontaneously within 3 months, but ∼30–40% of children have recurrent OM, and 5–10% of episodes last 1 year or longer, leading to a delay in learning and language development.^2^, ^3^, ^4^, ^5^

Approximately 29–50% of all URTIs develop into OM, particularly within two age ranges: 6 months–2 years of age and 5–6 years old. The main reasons for the young-age preference include: poorly developed immune defense, shorter and more horizontal Eustachian tube, well-endowed with lymphoid follicles and adenoids.^6^

Eustachian tube connects the tympanic cavity with the nasopharynx, playing a primary role in the defense mechanism of the middle ear and in the equilibration of its pressure with the atmospheric one; in addition, it protects the middle ear from reflux of nasopharyngeal secretions and drains secretions from the middle ear into the nasopharynx.^7^ Thus middle ear is an anatomical extension of the airway by way of the Eustachian tube and, as hypothesized by Nguyen et al., is considered a component of the “united airway concept”.^8^ Viral URTI leads to Eustachian tube inflammation resulting in its dysfunction and negative middle ear pressure permitting secretions containing the infecting virus and pathogenic bacteria that colonize the nasopharynx to enter the middle ear.^9^

## Objective

Environmental, epidemiologic and familial risk factors for OM (such as sex, socioeconomic and educational factors, smoke exposure, allergy or duration of breastfeeding) have been previously reported,^2^, ^3^, ^4^, ^5^, ^8^, ^10^, ^11^ but actually no data about their diffusion among Sicilian children with URTIs are available; thus, because of the high prevalence (14.43%) of OM in Sicilian children affected by URTIs,^4^ we examined the main OM risk factors, either separately or in combination, to provide additional data about their impact in the pathogenesis of OM.

## Methods

### Study design and population

This study was carried out by the Section of Audiology of the University of Palermo from September 2012 to June 2013, including 204 children (age range 2–10 years), 106 males and 98 females (M/F ratio = 1.08), who were examined for suspected URTI at the ENT pediatric ambulatory; all patients developed OM during a 3-week monitoring period. In addition, 204 age and sex-matched healthy children served as controls. The protocol of the study was approved by the local ethical committee (approval number V5604) and an informed consent was obtained from the parents of the children before enrollment in the study. There are no dropouts in the study. Patients and controls were subjected to a full history taking and complete physical examination including otoscopy and tympanometry.

The criteria for diagnosis of URTIs in the study were as follows: purulent nasal discharge, cough, pharyngo-tonsillar erythema or exudates.^11^

The criteria for diagnosis of OM (AOM and OME) in the study were: (1) AOM: acute onset of symptoms (fever, irritability, or earache) and signs (presence of fluid level, bubbles, hypervascularity, retracted tympanic membrane) of eardrum inflammation, as documented by pneumatic otoscopy and/or tympanometry^12^; (2) OME: documented middle ear effusion and/or air fluid bubble by otoscopic examination in presence of B or C tympanograms and, a conductive hearing loss greater than 25 dB at any one of the frequencies from 250 Hz through 4 kHz.^4^

OM was considered an URTI complication if it occurred within 21 days after the onset of URTI.^13^

### Risk factors and instrumental tests performed

Data were collected using a specific questionnaire answered by the parents about the gender, age, family members, educational status of the parents, exposure to smoking (yes; no), location of the residence, presence of pets, presence of air conditioning and/or mold, breastfeeding duration, atopic familiarity, age schooling, presence of allergy and/or asthma, presence of recurrent URTIs with OM (≤5 episodes; ≥6 episodes), cough and runny nose. In addition, questions about whether the child had difficulty breathing during sleep, mouth breathing and snoring were included; episodes of apnea were documented through modified portable sleep apnea monitoring, with recording of abdominal and chest movements, body position, snoring, oxygen blood saturation, pulse rate, oronasal airflow (nasal air pressure). Some children were affected also by pathological laryngopharyngeal reflux disease (LPRD), documented by a twenty-four hour double-probe pH-metry. This pH-monitoring device consisted of a proximal (2 cm above upper esophageal sphincter) and a distal probe (3 cm above lower esophageal sphincter); a laryngopharyngeal reflux episode was defined as a decrease in the pH level to lower than 4 for a duration of 15–30 s, measured at the proximal probe immediately following distal esophageal acid exposure without eating or swallowing.

At the time of the first appointment, all patients underwent skin tests. These were performed using skin prick tests for 12 common perennial and seasonal allergens: *Alternaria*, *Aspergillus*, *Cladosporium*, *Penicillium*, ragweed, grass mix, trees mix, cockroach, dust mites, *Dermatophagoides farinae* and *Dermatophagoides pteronyssinus*, and cat and dog epithelium.

The results were evaluated after 10 min. Wheals ≥3 mm in diameter than wheals at the site of the negative control were considered positive.

Cases and controls were subsequently included in the case–control study to establish the role of various risk factors for OM.

### Statistical methods

Statistical analysis was conducted with Matlab^®^ computer program. We performed partial logistic regression coefficient (b), logistic Odds Ratio (OR), 95% Confidence Interval (95% CI), multivariate logistic regression analysis and Mantel Haenszel test (Global Odds Ratio, G.*or*) to study the association between risk factors and OM.

## Results

We enrolled 408 children, 204 cases and 204 controls, with age range from 2 to 10 years (mean age of 5.56 ± 3.30 years).

Table 1 describes the distribution of each demographic, parental and environmental risk factor among cases (Group A) and controls (Group B). The 86.2% of the Group A was characterized by a medium or a low parental educational attainment with respect to the 70.6% of the Group B (*p* = 0.0001).

**Table 1:** Distribution of OM risk factors in cases and controls groups: chi square (*χ*^2^), Odds Ratio (OR), *p*-value and 95% Confidence Limits (95% CI).

Risk factor	Cases (Group a) *n* (%)	Controls (Group b) *n* (%)	OR	*χ*^2^	*p*-Value	95% CI
*Family size*						
0–3	52 (25.5)	18 (8.8)	3.54	19.9	0.0001	1.98–6.30
4–6	152 (74.5)	186 (91.2)				

*Parental educational attainment*						
No	4 (1.9)	–	–	71.9	0.0001	–
Low	68 (33.3)	116 (56.8)				
Medium	104 (50.9)	28 (13.7)				
High	28 (13.7)	60 (29.4)				

*Smoke exposure*						
Yes	72 (35.3)	38 (18.6)	2.38	14.38	0.0001	1.51–3.75
No	132 (64.7)	166 (81.4)				

*Residence*						
Urban	156 (76.5)	134 (65.7)	1.70	5.77	0.0162	1.10–2.62
Rural	48 (23.5)	70 (34.3)				

*Presence of pets*						
Yes	52 (25.5)	26 (12.7)	2.34	10.71	0.0011	1.39–3.93
No	152 (74.5)	178 (87.2)				

*Presence of air conditioning*						
Yes	124 (60.8)	166 (81.4)	0.35	21.03	0.0001	0.23–0.56
No	80 (39.3)	38 (18.6)				

*Indoor exposure to mold*						
Yes	84 (41.2)	26 (12.7)	4.79	41.87	0.0001	2.92–7.88
No	120 (58.8)	178 (87.2)				

*Breast-feeding*						
Yes	124 (60.8)	154 (74.5)	0.50	10.16	0.0014	0.33–0.77
No	80 (39.2)	50 (24.5)				
Never	80 (39.2)	52 (25.5)		8.78	0.012	
<4 months	72 (35.3)	88 (43.1)				
5–9 months	52 (25.5)	64 (31.4)				

*Atopic familiarity*						
Yes	92 (45.1)	66 (32.3)	1.72	6.98	0.0087	1.15–2.57
No	112 (54.9)	138 (67.6)				

*Age schooling*						
Yes	180 (83.2)	182 (89.2)	0.91	0.098	0.7542	0.49–1.68
No	24 (11.8)	22 (10.8)				
0–1	36 (17.6)	36 (17.6)		8.101	0.010	
2–3	152 (74.5)	132 (64.7)				
4–6	16 (7.8)	36 (17.6)				

*Allergy*						
Yes	32 (15.7)	34 (16.6)	0.93	0.072	0.7880	0.55–1.58
No	172 (84.3)	170 (83.3)				

*Asthma*						
Yes	16 (7.8)	2 (0.9)	8.06	11.39	0.0007	1.95–37.9
No	188 (92.1)	202 (99.0)				

*OM recurrences per year*						
<5	68 (33.3)	194 (95.1)	0.03	169.33	0.0001	0.01–0.05
>5	136 (66.6)	10 (4.9)				

*Snoring*						
No	92 (45.1)	174 (85.3)	7.06	72.63	0.0001	4.39–11.4
Yes	112 (54.9)	30 (14.7)				

*Apnea*						
No	76 (37.2)	30 (14.7)		81.24	0.0001	
Yes	36 (17.6)	–				

*Cough*						
Yes	148 (72.5)	86 (42.1)	3.63	38.51	0.0001	2.40–5.49
No	56 (27.4)	118 (57.8)				

*Runny nose*						
Yes	180 (88.3)	78 (38.2)	12.12	109.71	0.0001	7.3–20.2
No	24 (11.8)	126 (61.7)		2		
Serous	40/180 (22.2)	37/78 (47.4)		51.4	0.0001	
Mucous	60/180 (33.3)	41/78 (52.6)				
Seromucous	80/180 (44.4)	–				
Perennial	60/180 (33.3)	5/78 (6.4)	7.30	20.9	0.0001	2.8–19.1
Seasonal	120/180 (66.6)	73/78 (93.6)				

*LPRD*						
Yes	16 (7.8)	–	17.36	13.88	0.0002	2.2–132.2
No	188 (92.2)	204 (100)				

The 15.7% (32/204) of cases had positive skin tests for inhalant and food allergens whereas the 16.6% (34/204) of controls resulted atopic (*p* = 0.78); statistical analysis showed a significant difference between the groups regarding the prevalence of asthma, with a higher percentage of asthmatic children in the Group A (7.8%) respect to Group B (0.9%) (*p* = 0.0007).

No difference between cases and controls were found according to “age schooling”; however the 92.1% (188/204) of cases and the 82.3% (168/204) of controls were schooled within the third year of life.

The 26.9% of the total children studied were exposed to smoke, with a prevalence of this risk factor found in the 35.3% of Group A respect to the 18.6% of Group B (*p* = 0.0001).

Concerning the “residence” (urban or rural) different frequencies were found in the Group A (76.5% of urban vs. 23.5% of rural location) and in the Group B (65.7% of urban vs. 34.3% of rural residence) (*p* = 0.01).

Among the other variables examined in the univariate analysis, presence of pets (*p* = 0.001) and indoor exposure to mold (*p* = 0.0001) resulted strictly correlated to OM.

The 60.8% of Group A was breastfed instead of the 74.5% of the controls (*p* < 0.05). A significant difference in the “breastfeeding duration” was observed between these groups (*p* = 0.01).

“Snoring”, “cough”, “runny nose” and “LPRD” were found respectively in the 54.9%, 72.5%, 88.3% and 7.8% of group A, and the 14.7%, 42.1%, 38.2% and 0% of Group B (*p* < 0.05). A statistical difference in the prevalence of “atopic familiarity” was evidenced between the groups (45.1% for cases, 32.3% for controls) (*p* = 0.0087).

The study of recurrences showed that the 66.6% of cases presented more than 5 episodes of OM per year respect to the 4.9% of controls (*p* < 0.001). From the univariate logistic regression analysis it was evidenced a higher correlation between “recurrence of OM” and low parental educational attainment (*p* < 0.05) and runny nose (*p* < 0.01).

## Discussion

URTI and OM are diseases often associated in children. From 6% to 64% of patients affected by URTI develop OM, with the prevalence varying considerably by the age group on which the estimates are based.^2^, ^5^ Koch et al.^13^, studying 288 children, evidenced a higher risk among children aged 6–23 months than among children aged 0–5 months; Rupa et al.^14^ demonstrated that URTIs started within a few weeks of life with a progressive increase in frequency and a peak of 72% in the 9th month. Revai et al.^5^ reported a prevalence of 36% of OM in URTI infants aged between 6 and 11 months old and of 29% among children in the second year of life while, by increasing age, the prevalence of OM decreased. Also our data, with the 67% of cases aged 2–5 years, suggest the higher susceptibility of younger children to develop OM.^4^, ^5^, ^15^

Rupa et al.^14^, in line with our results (OR = 1.97; 95% CI: 1.13–3.44; *p* < 0.05), found that OM occurred more frequently in boys, differently from Koch et al.^13^ and Engel et al.^15^, who evidenced a higher prevalence among girls but without any statistical difference. However it is possible, according to Tos et al.^16^ and to Saim et al.^17^, that gender could represent a confounding factor and that results could be influenced by cultural factors or other infection diseases.

Koch et al.^13^ and Zielhuis et al.^18^ reported a higher OM prevalence in the upper socioeconomic classes while, on the contrary, other authors reported that this disorder was more common in presence of a lower socioeconomic status.^13^, ^14^, ^19^ Our findings showed that a lower parental educational level was associated with a higher percentage of OM and that OM recurrences were infrequent when the parents had a high educational attainment. In fact, in the 86.27% (176/204) of cases, parents had a low or medium educational level respect to the 70.6% (144/204) of controls (*p* < 0.001)

According to Martines et al.^4^ and Koch et al.^13^, “large family” is not a risk factor for OM. In fact, the 82.8% (338/408) of our cohort, the 74.5% of Group A and the 91.2% of Group B belong to families with more than 4 members (*p* = 0.0001).

With a percentage rate of 60.8% (124/204) of “presence of air conditioning” among children with OM (*p* = 0.0001), air conditioning may be a protective factor, although “WHO Guidelines for Indoor Air Quality: Dampness and Mould”^20^ affirm that in residential buildings and in hot climates, such as Sicilian one, air-conditioning can introduce excess humidity, chemicals (used to treat the water in humidification systems) or microorganisms promoting respiratory infections. We found a high OM prevalence (41.2%) among the 110 children exposed to mold (*p* = 0.0001). In line with our data, Pettigrew et al.^21^ recently demonstrated a close relationship between OM and indoor exposure to mold in 806 infants (OR = 3.45; 95% CI: 1.36–8.76).

According to Bergroth et al.^22^, who evidenced that pet contacts during infancy may have a protective effect on respiratory tract symptoms and infections, we observed that OM episodes per year were less frequent among children who have been living with pets from the early childhood (*p* < 0.01). Therefore animal contacts could help to mature the immunologic system, leading to more composed immunologic response and shorter duration of infections.

Because of asthma is usually associated with rhino-sinusitis (allergic, non-allergic or infective),^23^ it could be considered an “alarm bell” of a flogistic event in different sides of respiratory tract^16^, ^24^; in this study, the 7.8% of asthmatic subjects presented OM within 3 weeks from first examination (*p* < 0.05). It is also confirmed by multivariate analysis that evidenced in atopic children an increase of 11% of the relative risk of OM in the presence of asthma and pets (Table 2).

**Table 2:** Cases: allergic vs. non-allergic. Multivariate logistic regression analysis of OM recurrences.

	OM episodes per year										
Risk factors	<5 episodes					>5 episodes					Global Odds Ratio (G. *or*)
	Allergy					Allergy					95% Confidence Limits (95% CI)
	Yes		No		Total	Yes		No		Total	
	*n*	%	*n*	%		*n*	%	*n*	%		*p*-Value
*Asthma*											
Yes	2	0.9	6	2.9	8	6	2.9	4	1.9	10	G. *or* = 1.11
No	10	3.9	52	25.5	60	18	8.8	108	52.9	126	95% CI: 0.37–12.66
Total	12	4.9	58	28.4	68	24	11.7	112	54.8	136	*p* = 0.0001

*Presence of pets*											
Yes	2	0.9	14	6.8	16	2	0.9	34	16.6	36	
No	8	3.9	44	21.5	52	20	9.8	80	39.2	100	
Total	10	4.9	58	28.4	68	22	10.8	114	55.8	136	

*Cough*											
Yes	8	3.9	34	16.6	42	22	10.8	82	40.2	104	G. *or* = 1.74
No	2	0.9	24	11.7	26	2	0.9	30	14.7	32	95% CI 0.55–2.91
Total	10	4.9	58	28.4	68	24	21.5	112	54.9	136	*p* = 0.0001

*Runny nose*											
Yes	10	4.9	50	24.5	60	26	12.7	82	40.2	108	
No	2	0.	8	3.9	10	6	2.9	20	9.8	26	
Total	12	5.8	58	28.4	70	32	15.6	102	50.0	134	

From the analysis of the joint effect of risk factors, it resulted that smoke exposure increases the risk of OM of 277% and of 166% when in presence respectively of urban localization and allergy (Table 3). These results supported Nguyen et al. hypothesis, that especially in children where the mucociliary clearance and the anatomy of the Eustachian tube are still abnormal the joint effect of risk factors can increase exponentially the recurrence of OM.^8^

**Table 3:** Cases: smoke exposure vs. no smoke exposure. Multivariate logistic regression analysis of OM recurrences.

	OM episodes per year										
Risk factors	<5 episodes					>5 episodes					Global odds ratio (G. *or*)
	Smoke exposure					Smoke exposure					95% Confidence Limits (95% CI)
	Yes		No		Total	Yes		No		Total	
	*n*	%	*n*	%		*n*	%	*n*	%		*p*-Value
*Residence*											
Urban	22	10.8	38	18.6	60	58	28.4	38	18.6	96	G. *or* = 3.77
Rural	2	0.9	6	2.9	8	10	4.9	30	14.7	40	95% CI= 2.01–10.44
Total	24	11.7	44	21.5	68	68	33.3	68	33.3	136	*p* = 0.0002

*Allergy*											
Yes	4	1.9	12	5.8	16	12	5.8	4	1.9	16	G. *or* = 2.66
No	12	5.8	40	19.6	52	44	21.5	76	37.2	120	95% CI= 1.57–17.05
Total	16	7.8	52	25.5	68	56	27.4	80	27.4	136	*p* = 0.017

The role of breastfeeding for the protection of infant is universally accepted; scientific researches, such as the studies summarized in a 2007 review for the U.S. Agency for Healthcare Research and Quality (AHRQ) and a 2007 review for the WHO, have found many benefits of breastfeeding in prevention of URTIs, severe lower respiratory tract infections, non-specific gastroenteritis, and OM.^25^, ^26^ Our results agree with these studies and with those of Teele et al.^27^ and Zielhuis et al.^21^, showing that the 71.6% of total children (292/408) was not breast-feed or breast-fed for less than 4 month; specifically the 39.2% (80/204) of children who developed OM were not breast-feed (*p* = 0.0014; OR = 10.16).

Habitual snoring (HS) and apneas which were found respectively in the 37.2% (76/204) of cases and the 14.7% (30/204) of controls were epidemiologically linked to many of the same risk factors that have been identified for OM. According to Caylan et al.^28^ and Li et al.^29^, who concluded that the presence of snoring is correlated with a higher prevalence of OM, we evidenced that the 37.2% of Group A and the 14.7% of the control group affected by snoring and/or apnea (*p* = 0.0001).

Of the 146 children with a positive history of more than 5 episodes per year of URTI with OM, the 93.1% were affected by OM at our examination (*p* < 0.05); the risk of OM recurrences increase of 74% in presence of allergy, persistent cough and runny nose (*p* = 0.0001) (Table 2). These data confirmed, as reported from “Panel report from the Ninth International Research Conference on Otitis Media”,^30^ that OM is frequently a complication of URTI and a history of frequent episodes of OM and URTI is itself a risk factor because increases host susceptibility to respiratory tract infections.^31^

With a percentage of 7.8% (16/204) among children with OM (*p* = 0.0002), LPRD may be a risk factor for URTI complications because of the mucosal inflammation that obstructs the Eustachian tube.^32^

## Conclusions

URTIs and OM are multifactorial diseases common during childhood. This paper contributes in the understanding of the role of different risk factors in the development of OM among children affected by URTIs. Specifically, our data supported parental educational attainment (*p* = 0.0001), exposure to smoke (*p* = 0.001), indoor exposure to mold (*p* = 0.0001), lack of breast-feeding (*p* = 0.0014) and LPRD (*p* = 0.0002) as main risk factors for OM; additionally children affected by cough, runny nose, asthma or snoring were more susceptible to develop OM. Finally, an increased risk (74%) of OM recurrences was observed in the presence of allergy, persistent cough and runny nose (*p* = 0.0001). Comprehensive knowledge of modifiable risk factors found in this study could contribute to minimizing URTI and its complications in children.

## Conflicts of interest

The authors declare no conflicts of interest.
